# Retinal Disease Detection Using Deep Learning Techniques: A Comprehensive Review

**DOI:** 10.3390/jimaging9040084

**Published:** 2023-04-18

**Authors:** Stewart Muchuchuti, Serestina Viriri

**Affiliations:** School of Mathematics, Statistics and Computer Science, University of KwaZulu-Natal, Durban 4001, South Africa

**Keywords:** macula degeneration, convolutional neural networks, diabetic retinopathy, hypertensive retinopathy, deep learning, glaucoma, retinal disease classification

## Abstract

Millions of people are affected by retinal abnormalities worldwide. Early detection and treatment of these abnormalities could arrest further progression, saving multitudes from avoidable blindness. Manual disease detection is time-consuming, tedious and lacks repeatability. There have been efforts to automate ocular disease detection, riding on the successes of the application of Deep Convolutional Neural Networks (DCNNs) and vision transformers (ViTs) for Computer-Aided Diagnosis (CAD). These models have performed well, however, there remain challenges owing to the complex nature of retinal lesions. This work reviews the most common retinal pathologies, provides an overview of prevalent imaging modalities and presents a critical evaluation of current deep-learning research for the detection and grading of glaucoma, diabetic retinopathy, Age-Related Macular Degeneration and multiple retinal diseases. The work concluded that CAD, through deep learning, will increasingly be vital as an assistive technology. As future work, there is a need to explore the potential impact of using ensemble CNN architectures in multiclass, multilabel tasks. Efforts should also be expended on the improvement of model explainability to win the trust of clinicians and patients.

## 1. Introduction

A compromise of human vision quality adversely affects one’s productivity and general quality of life. Millions of people are affected by retinal abnormalities worldwide and, if not diagnosed and treated early, may result in vision loss [1,2]. Early detection and effective treatment of retinal diseases could arrest the further progression of the diseases and possibly save multitudes from losing vision. Retinal diseases include Choroidal Neovascularization (CNV), Age-Related Macular Degeneration (AMD), Diabetic Macula Edema (DME), glaucoma, Drusen and diabetic retinopathy (DR) [3]. Tamim et al. [4] predicted that the number of people with glaucoma will peak to 111.8 million people by 2040. According to Chelaramani et al. in [5] in 2015, 415 million people were suffering from diabetes, of which 145 million had DR. Chelaramani et al. [5] further stated that AMD affects 6.2 million people globally.

Experienced ophthalmologists make use of retinal images captured by either fundus cameras or Optical Coherence Tomography (OCT) to detect the presence or absence of each of the retinal diseases. This manual process, as observed by Qummar et al. in [6], is time-consuming, tedious and subjective, making the reproducibility of such diagnoses hard to achieve. Access to medical specialists and infrastructure is limited in underdeveloped countries, especially in the countryside. This creates room for the automatic detection of retinal diseases, provided the detection accuracies match or surpass human experts’ accuracy and are acceptable to the Food and Drug Associations (FDAs) of host countries. Automatic detection and grading of retinal diseases could also come in handy as assistive technology to alleviate the burden of the few overstretched ophthalmologists around the globe.

Computer-Aided Diagnostic (CAD) systems have been proposed lately, mostly to diagnose DR and AMD, taking advantage of the advances in Machine Learning (ML) and digital image processing. As observed by Jain et al. in [1], these Machine Learning-inspired medical diagnosis methods, examples of which are Decision Trees in [7] and the Gaussian Mixture Model in [8], managed to reach the accuracy levels of human experts, but their drawback was that they relied heavily on understanding the disease-specific features and took considerable effort to be able to extract, analyze and engineer the disease features.

Recently, deep learning (DL), a branch of ML, has been used with promising results for detecting specific retinal diseases [9]. There have been some considerable advances in the application of DL in the detection and grading of individual ocular disorders, for example, DR, AMD, DME and others, through the use of classification methods or through segmentation or a combination of the two techniques [10]. The success of transformer networks in natural language processing have led to attempts to apply them to computer vision, in general, and in retinal disease detection, in particular, with studies by [11,12,13] making significant contributions to model performance and model explainability.

Retinal image analysis involves the processing of images captured by fundus cameras, fluorescein angiography or Optical Coherence Tomography (OCT). As suggested by Gour and Khanna in [14], Fundoscopy and OCT imaging have emerged as the most popular non-invasive methods for capturing retinal morphological changes, such as optic disc, blood vessels, macula and fovea. Analysis of these images helps detect diseases such as DR, glaucoma, AMD, myopia, hypertension and cataract. There is a plethora of published work focusing on the detection of single diseases, notably DR, glaucoma and AMD [6,15]. As observed in [15,16,17,18,19], Deep CNNs and other Deep Neural Networks have been successfully used to develop Artificial Intelligence (AI) systems for the purposes of automated CAD, leveraging large clinical databases.

Contributions: Presented in this work is a comprehensive, in-depth review of recently published research aimed at improving the efficacy of DL techniques in the detection of retinal pathologies. Common ocular pathologies that are the focus of discussion in this work are reviewed in Section 2, while Section 3 outlines retinal imaging modalities commonly adopted for the detection of the same pathologies. Section 4 reviews the databases that have been commonly used for retinal disease classification purposes. A critical review of the retinal disease detection research is presented in Section 5 and Section 6, providing a discussion on the challenges inherent in DL approaches. Section 7 presents the final conclusion.

Scope of the article: Studies that involve the detection of the most prevalent ocular disorders, such as DR, DME, AMD and glaucoma, were considered. The focus was to analyze research that made attempts to apply DL architectures to detect present anomalies among the rest. Only DL methods, namely CNNs and ViTs, and their variants were considered for analysis. Traditional image analysis techniques, segmentation techniques and feature-based methods inspired by ordinary ML methods were not part of this study. Search queries were performed on Google Scholar and PubMed with keywords such as deep learning, Classification, Ophthalmology, Medical Image Datasets, deep learning in Ophthalmology, Fundoscopy, and OCT Imaging.

## 2. Common Ocular Disorders

This section examines the morphology and anatomy of the retina and discusses the most prevalent retinal abnormalities, including DR, DME, AMD, CNV, glaucoma and cardiovascular disease. The section proceeds to discuss the imaging modalities commonly used for detecting and grading retinal diseases. DR, AMD and glaucoma were the abnormalities of interest in this study.

### 2.1. Structure of the Eye

**Figure 1:**
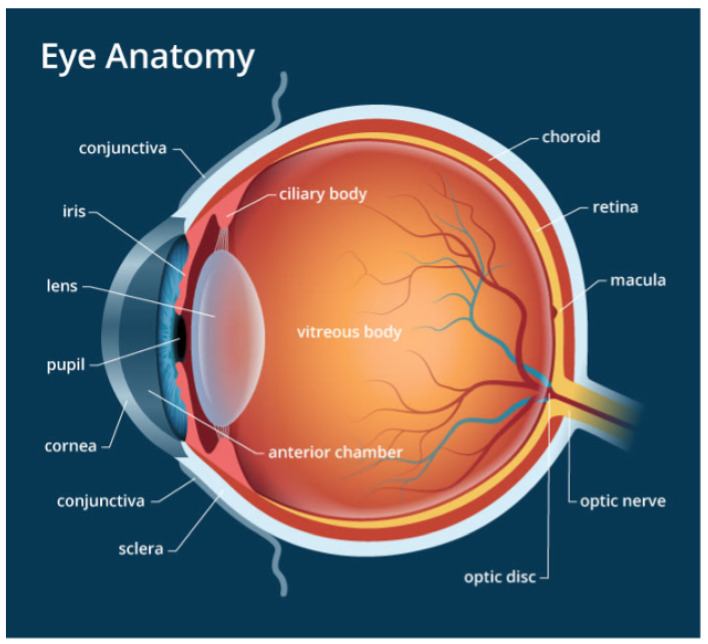
The Structure of the Eye.

**Figure 2:**
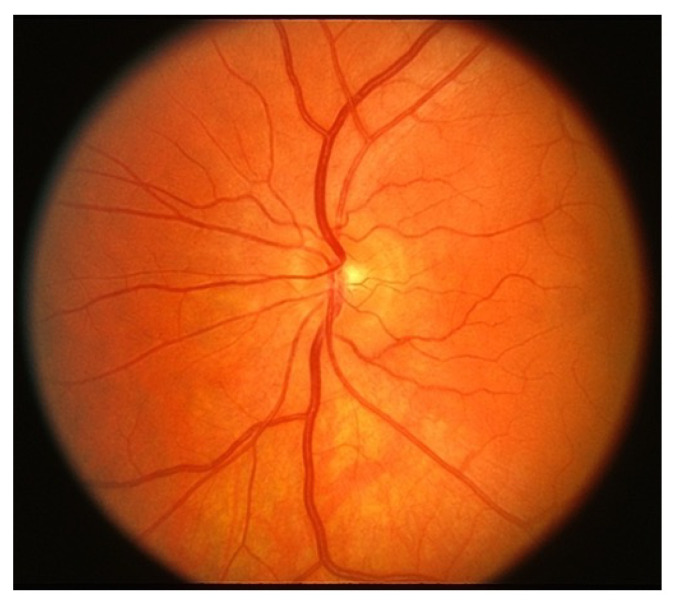
Normal Retina.

Easily identifiable components of the human eye include the sclera, cornea, iris and pupil. The interior surface consists of the retina, macular, fovea, optic disc and posterior pole, as depicted in Figure 1. When humans look at an object, light flushes the cornea, which partially focuses the image before it is passed to the pupil and then the lens. The lens further focuses the image. The image is then passed through the vitreous before being focused on a portion of the central retina named the macula [20]. This specialized portion of the retina allows humans to see fine detail for activities such as reading, writing and distinguishing colors. The other part of the retina, the peripheral retina, is responsible for side vision. The retina, a layered tissue in the eye, has the responsibility of converting light incident on it into a neural signal passed on to the brain for further analysis [21]. This makes the retina an extension of the brain. Blood supply to the retina is through a network of blood vessels. Diseases such as diabetes have the tendency to damage blood vessels of the retina and in the process, disrupt its operation. Figure 2 shows the image of a normal retina taken by a fundus camera.

### 2.2. Systemic Diseases Manifesting in the Retina

A plethora of diseases emanating from the eyes, the brain or the cardiovascular system manifest themselves through the retina. This section discusses the most common pathologies that can be studied through retinal imaging.

#### 2.2.1. Diabetic Retinopathy (DR)

A patient recording a plasma glucose above 7.0 mmol/L is diagnosed to be having diabetic mellitus, according to the World Health Organization [22]. The presence of elevated blood glucose, called hyperglycemia, could potentially damage blood vessels and nerve cells, leading to damage to the kidneys, heart, brain and eyes. Complications on the retina caused by damage to the retinal vessel walls are called diabetic retinopathy (DR). Abramoff et al. in [21] suggests that DR is one of the leading causes of vision loss among adults.

**Figure 3:**
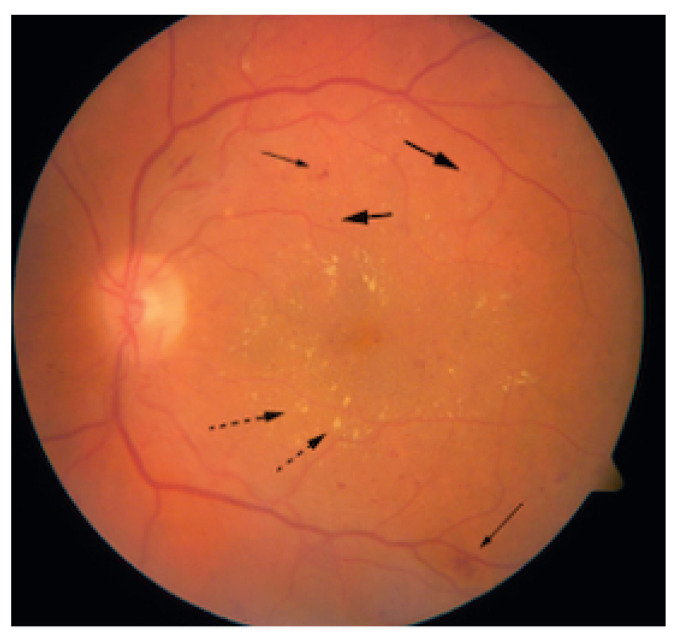
Retina with DME (Solid arrows: Microaneurysms, Dashed arrows: Exudates).

**Figure 4:**
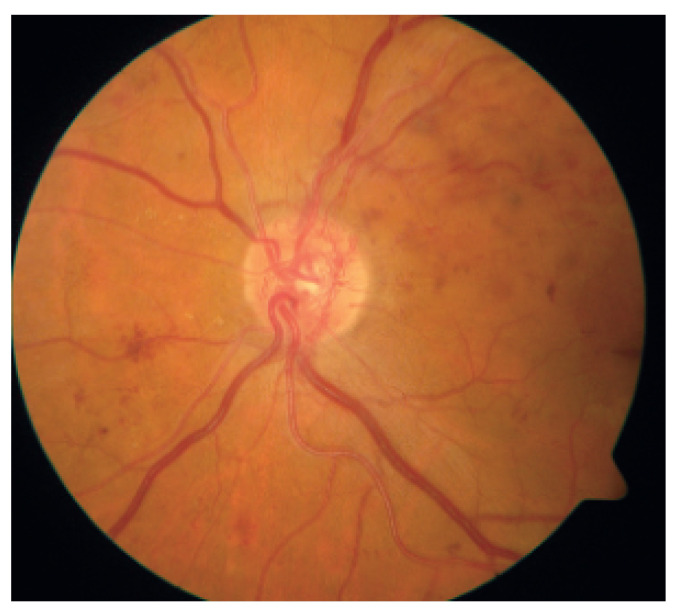
Proliferative Diabetic Retinopathy.

Damages to the retinal vessel walls due to hyperglycemia can lead to either of two conditions, ischemia or diabetic macula edema (DME). Ischemia is when new blood vessels emerge, and because they are weak, they may subsequently rupture, causing serious hemorrhages, which cause vision obstruction or even permanent loss of sight [21]. This condition, which is also called Neovascularization, leads to proliferative diabetic retinopathy. When the blood–retinal barrier breaks down, this leads to fluid leakage, which could affect central vision. This condition is called DME and can also be associated with the destruction of photoreceptors. DME is the major cause of vision impairment in people living with diabetes [23]. Figure 3 shows a fundus picture of the retina exhibiting DME, with evidence of hemorrhages, exudates and microaneurysms. Figure 4 is a depiction of the creation of new blood vessels leading to proliferative diabetic retinopathy.

#### 2.2.2. Age-Related Macular Degeneration (AMD)

**Figure 5:**
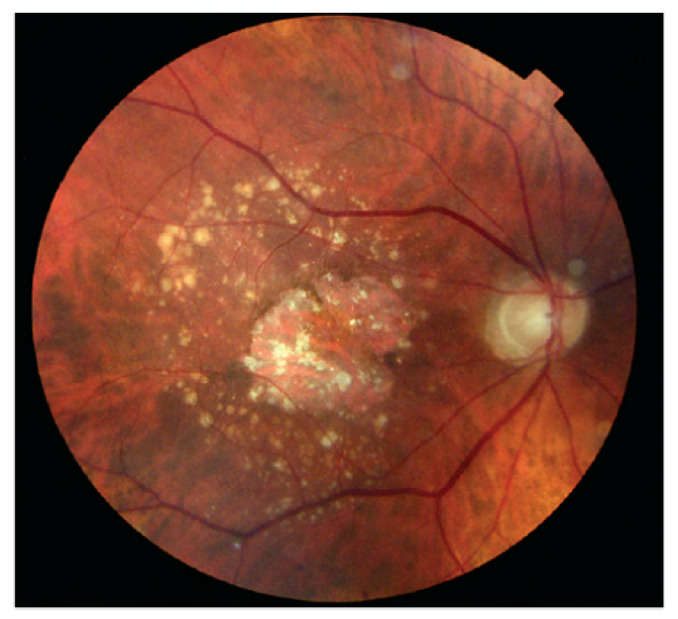
Age-Related Macular Degeneration.

AMD is the main source of loss of vision, accounting for 54 percent of all legally blind Americans [21]. AMD, prevalent in people of age 50 and above, is caused by the deterioration of the macula due to age. The estimated annual cost burden to the USA economy as a result of AMD is USD 30 billion [21]. The formation of Drusen (tiny yellow pieces of fatty protein) under the retina usually precedes AMD. The major categories of AMD are dry and wet AMD [20]. Vision impairment or loss is usually gradual with dry AMD. Wet AMD, also known as Choroidal Neovascularisation (CNV), is the most sight-threatening type of AMD. A retinal image exhibiting AMD is shown in Figure 5.

#### 2.2.3. Glaucoma

Glaucoma, which is primarily a neuropathy and not retinopathy, is caused by destruction of the optic nerve. This, in turn, results in visual field loss. Glaucoma damages the ganglion cells and axons of the retina [21]. This happens when the eye fluid, called the aqueous humor, does not circulate properly in the front end of the eye. Optic disc cupping is the hallmark of glaucoma. It is the visual exposition of the optical nerve head (ONH) anatomy. Glaucoma is the third leading cause of visual loss, according to [21].

#### 2.2.4. Cardiovascular Disease

The presence of cardiovascular disease becomes evident in the retina, mostly through hypertension and atherosclerosis. These usually result in a decrease in the Artery to Vein (A/V) ratio as arteries thin out and veins widen. The changes to the A/V ratio usually lead to an increased risk of myocardial infarction and stroke [24,25].

#### 2.2.5. Hypertensive Retinopathy

This is a condition affecting retinal blood vessels. Elevated blood pressure can lead to damage of the retinal vessels. This could lead to swelling, bleeding and damage of the optic nerve. Blurred vision, headaches and double vision are among the signs a patient suffers from hypertensive retinopathy. This pathology, as reported by [26], is highly progressive, affects men more than women and impacts 4–18 percent of the general population.

#### 2.2.6. Cataracts

Cataracts are a common retinal disease characterized by the clouding of the eye lens, leading to a deterioration in vision. It is a leading cause of visual impairment and avoidable blindness worldwide, particularly in older adults. The development of cataracts is associated with aging, as well as other factors, such as smoking and exposure to ultraviolet light [27].

Cataracts are typically diagnosed through a comprehensive eye exam, which may include visual acuity testing, tonometry to measure intraocular pressure and a dilated eye exam to examine the lens and other structures of the eye. Treatment for cataracts typically involves surgery to remove the cloudy lens and replace it with an artificial lens implant [28].

There are various types of cataracts, including age-related cataracts, congenital cataracts, traumatic cataracts and secondary cataracts associated with other medical conditions or medications. The classification of cataracts is based on the location and appearance of the clouding within the lens.

#### 2.2.7. Retinal Detachment

Retinal detachment is a serious ocular condition in which the retina becomes separated from its normal position. This detachment disrupts the blood supply to the retina, leading to vision loss and potential blindness if left untreated. The main symptoms of retinal detachment include sudden onset of floaters, flashes of light and a curtain-like shadow over the field of vision [29].

The causes of retinal detachment include aging, trauma to the eye and underlying medical conditions such as diabetes. Treatment for retinal detachment typically involves surgery, such as scleral buckling or vitrectomy, to reattach the retina and restore vision [30].

#### 2.2.8. Macular Edema

Macular edema is a medical condition characterized by the accumulation of fluid in the macula, which is the central part of the retina responsible for sharp, clear vision. It can occur as a result of various conditions, including diabetic retinopathy, Age-Related Macular Degeneration and retinal vein occlusion. Macular edema can cause blurry or distorted vision, and if left untreated, it can lead to permanent vision loss.

Treatment for macular edema may include medication, such as corticosteroids or anti-VEGF drugs, laser therapy or surgery, depending on the underlying cause and severity of the condition [31,32].

#### 2.2.9. Retinopathy of Prematurity

Retinopathy of prematurity (ROP) is a disease that affects premature infants and is characterized by abnormal blood vessel growth in the retina, which can lead to vision loss or blindness if left untreated. It is a leading cause of blindness in children worldwide.

The development of ROP is associated with premature birth and low birth weight, as well as other factors, such as oxygen therapy and certain medical conditions. ROP is typically diagnosed through a comprehensive eye exam that may include dilated fundus examination and imaging tests, such as retinal photography or Optical Coherence Tomography.

Treatment for ROP depends on the severity of the disease and may include monitoring the disease, laser therapy or surgery. Early detection and treatment are important to prevent vision loss [33,34].

#### 2.2.10. Refractive Errors

Refractive errors are a group of common vision disorders that affect the ability of the eye to focus on objects at different distances. These conditions are caused by abnormalities in the shape or size of the eye or the curvature of the cornea, which prevent light from being properly focused on the retina. The most common types of refractive errors include myopia (nearsightedness), hyperopia (farsightedness), astigmatism and presbyopia (age-related farsightedness).

Myopia is a condition where distant objects appear blurry while near objects are seen clearly. Hyperopia is the opposite, with distant objects appearing clearer than near objects. Astigmatism occurs when the cornea or lens has an irregular shape, causing blurred vision at all distances. Presbyopia is a condition that affects people over the age of 40, and it results in the gradual loss of the eye’s ability to focus on close objects.

Refractive errors can be diagnosed through a comprehensive eye exam that includes a visual acuity test and a refraction test. Treatment typically involves corrective lenses, such as glasses or contact lenses, or refractive surgery, such as Laser-Assisted in Situ Keratomileusis (LASIK) [35,36].

#### 2.2.11. Summary

Several systemic and eye diseases become manifested in the retina. This section outlines the different manifestations of retinal maladies that include DR, glaucoma and AMD. It was observed in [25] that some cardiovascular diseases can also manifest themselves in the retina. Early detection and treatment of these ocular disorders prevent complications, some of which can be severe, including visual impairment or permanent blindness.

## 3. Overview of Retinal Imaging Modalities

Having come through ages of constant research, retinal imaging has become the pillar of clinical management and care of patients with retinal and systemic diseases as observed by [21]. Persistent research on retinal imaging has led to improved visualization quality of retinal pathophysiology, which has resulted in early and more accurate diagnosis and better management of several chorio-retinal abnormalities. Fundus photography, Optical Coherence Tomography (OCT) and their variants have become the most prevalent retinal imaging modalities [23]. This section presents an overview of these two modalities, highlighting their suitability in various retinal disease diagnostic operations.

### 3.1. Fundus Imaging

Fundus imaging, as suggested by [23] is where a two-dimensional (2-D) representation of three-dimensional retinal tissues cast onto the imaging surface is attained using reflected light. The image intensities on the 2-D projection are proportional to the amount of light reflected from the retinal tissue. Variants of fundus imaging include scanning laser ophthalmoscopy, adaptive optics SLO, color fundus photography and hyperspectral imaging, among others. This section highlights some modalities that fall under fundus imaging.

#### 3.1.1. Fundus Autofluorescence

Fundus Autofluorescence (FAF) allows for the mapping of the retinal pigment epithelium and the photoreceptor layer in vivo [37]. Molecules are brought to glow through the excitation of light within a certain range of wavelengths. There is no need to inject any intravenous agent into the eye since the intrinsic molecules are already present. This imaging modality has proven to be useful in comprehending pathophysiological mechanisms, predictive marker identification and diagnosis. FAF finds applications in imaging toxic retinopathies, AMD and retinal tumors, among other abnormalities.

#### 3.1.2. Adaptive Optic Scanning Laser Ophthalmoscopy (AO-SLO)

The AO subsystem in AO-SLO consists of a liquid crystal spacial light modulator. It is this technology that gives the AO subsystem the ocular optics aberration compensation capabilities, which result in high image quality. A 780 nm laser diode is used as the light source for wavefront sensing. The light source for the SLO subsystem is an 840 nm super-luminescent diode (SLD). This modality produces images of high resolution through the use of custom software to control the image acquisition process. One big advantage of the AO-SLO is its capability to scan wider portions of the retina better than other modalities [38,39].

#### 3.1.3. Fundus Fluorescein Angiography (FFA)

FFA is a standard ground-truth imaging modality that has been applied in the diagnosis of many retinal diseases, including AMD and DR. High-resolution FFA images help detect small lesions, such as microaneurysms (MAs), early in their development. This could possibly help avert vision-threatening complications [40]. There are two challenges associated with FFA. Its invasive nature leads to an inherent small risk of anaphylaxis. Moreover, practical application constraints may lead to delays in FFA being performed. These delays may lead to delays in treatment, potentially affecting patient visual outcomes.

#### 3.1.4. Indocyanine Green Angiography (ICGA)

With ICGA, indocyanine green contrast dye is injected into the eye. This is followed by ICGA using a laser light source and a charge-coupled camera. ICGA provides real-time perfusion assessment with great resolutions and diagnostic sensitivities. Its intravenous nature tends to lower the patient safety profile, and as a result, this modality’s use becomes limited as clinicians tend to prefer non-invasive modalities [41].

#### 3.1.5. Limitations of Fundus Imaging

Fundoscopy presents a few challenges. The passage of the externally illuminated light into the retina and the retina-reflected light are limited by the small size of the pupil and the tiny diameter of the iris. The external light incident on the retina and the reflected light follow separate paths to avoid the elimination of image contrast. This makes the technical setup of fundus imaging technically challenging, making the equipment expensive and requiring operators of fairly high experience and expertise [23]. There have been improvements in fundus imaging that have significantly made it more accessible over the last few years. These improvements include a shift to digital imaging from film-based imaging. This makes fundus imaging more user-friendly to allow even non-ophthalmic photographers through the introduction of more standardized imaging protocols [21]. Fundoscopy is predominantly used for large-scale, population-based detection of DR, glaucoma and AMD [23].

### 3.2. Optical Coherence Tomography (OCT)

OCT, a non-invasive imaging technique used for acquiring 3-D volumetric images, has become the modality of choice for the examination of the retinal structure [42]. It uses waves to capture the cross-section image of the retina. Using OCT, the eye specialist can view each of the retina’s several layers, allowing them to estimate their thickness. OCT attempts to estimate the depth at which an individual backscatter started from by computing its flight time. Light flight times are longer for deeper tissue backscatters than those coming from shallower tissues. Interferometry is employed to measure the light flight times, owing to the minute flight time differences resulting from the small total thickness of the retina (between 300–500 µm). As noted by [21] there are three main OCT methods developed to attain A-scan for the required depth range of the tissue.

#### 3.2.1. Swept Source Encoded Frequency OCT

With this technique, the reference arm is not moved, but the light source is quickly modulated about its center wavelength. To estimate the correlogram for each center wavelength, a photo center is used. Application of the Fourier transform helps establish the depth of tissue scatters at the imaged spot.

#### 3.2.2. Time Domain OCT

Time domain OCT involves moving the reference arm mechanically to distinct points. This results in different reference arm light flight time delays. The number of A-scans per second is limited to a few thousand with time domain OCT, owing to the limitations inherent with mechanical arm movements.

#### 3.2.3. Spectrum Domain OCT

The operation of this modality is similar to the one for swept source OCT, except that it uses a broadband light source. With this modality, diffraction and a CMOS linear sensor are used to spectrally decompose the interferogram. Fourier transform is still applied in this technique to establish the depth of each scatter signal.

### 3.3. Modalities Performance Comparisons

Adaptive Optics scanning ophthalmoscopy (AO-SLO) is an imaging modality based on Adaptive Optics (AO) Scanning and has been growing in popularity over the years [43]. By reducing the effects of wavelength distortions, AO is able to improve the performance of an optical system. Table 1 provides a comparison of the performance of the AO-SLO against commonly used imaging modalities: fundus fluorescein angiography (FFA), indocyanine green angiography (ICGA), OCT and fundus autofluorescence (FAF). The major strength of the AO-SLO over the conventional modalities is its ability to scan wider portions of the retina.

### 3.4. Summary

Ophthalmic imaging has seen explosive growth in recent years. Current retinal imaging techniques have contributed immensely to our appreciation of the pathophysiology and treatment of retinal disorders. In this section, two main ocular imaging modalities, fundus imaging and OCT imaging, were discussed. As the retinal image quality improves, the sensitivity and specificity of ocular malady detection and/or grading improves. The improved capabilities of digital technology to acquire, edit, archive and transmit retinal images and continued collaborations in this area are set to further improve retinal imaging for the benefit of patient management. Areas of continuous improvement with retinal imaging include portable, functional imaging, cost-effective fundus imaging, longer wavelength OCT imaging and adaptive optics.

## 4. A Review of Retinal Image Databases

This section outlines the major public and private image databases that have been used to evaluate the performance of algorithms in the literature recently. These databases have a defined gold standard, making them suitable for evaluating algorithm performance. The databases include retinal images with DR, AMD, glaucoma, hemorrhages, neoplasms and hypertension, among others. The public and private databases are discussed as follows.

### 4.1. Public Databases

The increasing need to validate or train models has driven research groups to create their own databases and make them public [48]. The DRIVE and STARE open-access databases are two of the most widely used retinal databases, owing to the superior resolutions of their fundus images [49].

#### 4.1.1. DRIVE (Digital Retinal Image for Vessel Extraction)

This database was created to enable blood vessel segmentation comparative studies. DRIVE has 40 images from retinal photos of 400 human subjects with ages ranging from 25 to 90 years. This JPEG format database has 33 images without DR symptoms and 7 images exhibiting mild DR symptoms [50]. The DRIVE website facilitates researchers to share the performances of their vessel segmentation algorithms.

#### 4.1.2. STARE (Structured Analysis of the Retina)

STARE has 400 retinal images, 40 of which contain manually segmented blood vessels and artery/vein labeling. All images are labeled by specialists. The image data were compressed into PPM format [51]. Algorithms for optic nerve head (ONH) identification are also included. A total of 44 pathologies were detected and associated with 13 different abnormalities.

#### 4.1.3. ARIA (Automatic Retinal Image Analysis)

The ARIA database is a JPEG format database containing 450 images. The images are categorized into three categories: a healthy control group, another with AMD and a third group with DR. Two expert ophthalmologists were responsible for annotating the images [50].

#### 4.1.4. CHASEDB (Child Heart Health Study in England Database)

This database contains 28 manually segmented monochrome ground-truth images with a resolution of 1280 × 960 pixels. Retinal imaging was performed for more than 1000 children to establish the link between ocular vessel tortuosity and cardiovascular disease risk factors [48]. Image segmentation was performed by expert ophthalmologists [52].

#### 4.1.5. IMAGERET

This is a public database consisting of two parts: DIARETBD0 and DIARETDB1, both of which are saved in the PNG format. DIARETBD0 has 130 images (20 normal and 110 exhibiting DR signs). DIARETBD1 has 5 DR-infested images and 84 images showing signs of mild proliferative DR [53].

#### 4.1.6. MESSIDOR

Methods to Evaluate Segmentation and Indexing techniques in the field of retinal ophthalmology within the Scope of Diabetic Retinopathy (MESSIDOR) is a TIFF format database originally constructed to evaluate and compare segmentation algorithms designed to identify retinal lesions. It is a fully labeled database depicting the DR grade for each of the 1200 color fundus images [54]. It is one of the largest available databases (1200 images) for retinal images created to facilitate the development of CAD systems for DR.

#### 4.1.7. MESSIDOR-2

This database of 1200 high-quality images contains 21.7% referable diabetic retinopathy images, 10.6% vision-threatening diabetic retinopathy images with the rest of the images being normal. The database contains two images per subject and single images for each eye. Subjects passed through three centers during image capture. They were dilated at the first two centers, but when they get to the third center, they were not dilated. The mean age of the subjects was 57.6, with 57% being male [55]. The images were graded by three board-certified specialists according to the International Clinical Diabetic Retinopathy Severity scale (ICDR, 0–4).

#### 4.1.8. e-Ophtha

This database, designed for diabetic retinopathy screening, was funded by the French Research agency. The images were manually annotated by a specialist ophthalmologist and confirmed by another. The database contains two subsets-e-Ophtha MA (Microneurisms) and e-Ophtha EX (Exudates). The total number of images in this dataset is 434, all gradable. A total of 203 patients participated in this project, funded by a French hospital [56].

#### 4.1.9. DIARET DB1

DIARET DB1 is an 89-color fundus image standard database used to benchmark diabetic retinopathy detection algorithms. Of the 89 images, 84 contain at least mild non-proliferative signs of DR. Several specialists were involved in the annotation of the images. Each of the specialists attached a degree of confidence to their annotations. The degrees of confidence are averaged to reach the agreed grade of an image. The remaining five images have sight-threatening abnormalities. The images were captured from Kuopio University Hospital using several cameras [57].

#### 4.1.10. APTOS

The Asia Pacific Tele Ophthalmology Society (APTOS) dataset is a Kaggle dataset of 3662 images of different sizes that was captured with different cameras. It was constructed by the Aravind Eye Hospital in India. In this database, only the ground truth of the training set is publicly available. The images are classified into the five ICDR classifications. The dataset is highly imbalanced, with most of the images (1805) being normal. Only 183 images have severe Non-Proliferative Diabetic Retinopathy (NPDR) [58]. Because APTOS images were taken in a real-world environment, they exhibit variations due to different camera settings across centers.

#### 4.1.11. AREDS

Age-Related Eye Disease Study (AREDS) was a longitudinal study of up to 12 years. In this study, many patients’ AMD conditions were followed up for this period. The study included Geographic Antrophy cases, neovascular AMD cases and control patients. Left- and right-side retinal images of each patient were taken for the duration of the study. The images were graded for AMD severity by different eye specialists. During the study, some patients who had earlier exhibited mild AMD symptoms progressed to more severe AMD stages. This database has training, validation and test sets of 86,770, 21,867 and 12,019 images, respectively [59].

#### 4.1.12. ORIGA

Online Retinal fundus image dataset for Glaucoma Analysis and research (ORIGA) was developed for segmenting the optic cup and the optic disc by the Singapore Malay eye Research Institute (SERI). It is a publicly available database with 650 retinal images for providing the means for benchmarking segmentation and classification algorithms. It has 168 glaucomatous and 482 healthy images, each of resolution 3072 × 2048 pixels [60]. The images were collected between 2004 and 2007 and annotated by well-trained professionals. The subjects in this study were aged between 40 and 80 years [61].

#### 4.1.13. ACRIMA

The ACRIMA dataset was a culmination of a project founded by the Ministerio de Economica y Campettividad of Spain, a unit dedicated to the development of algorithms for ocular disease detection. The database has 705 images—396 glaucomatous and 309 normal. The images were captured using the Topcon TRC retinal camera from previously dilated left and right eyes. Image annotation was performed by two glaucoma experts with 8 years of experience [62].

#### 4.1.14. RIM-ONE

Retinal Image Database for Optic Nerve Evaluation (RIM-ONE) consists of 159 fundus images, each with a resolution of 2144 × 1424 pixels. All images have optic cup and optic disc annotations. Of the 159 images, 74 are glaucomatous and 85 are normal. Images from the three RIM-ONE versions were taken from three Spanish hospitals [63].

#### 4.1.15. LAG DB

The Large-Scale Attention Glaucoma (LAG) database, a collection of 11,760 images from 10,147 subjects, was created by the Chinese Glaucoma Study Alliance and the Beijing Tongren Hospital. Of all the images, 4878 exhibit positive glaucoma, while the remaining 6882 are normal [64]. The images have an average resolution of 1977 × 2594 pixels. Apart from the fundus image and the diagnosis label, the database also consists of an attention region to help localize the abnormalities. Several ophthalmologists with varying expertise were involved in the annotation process.

#### 4.1.16. OCT2017

The OCT2017 database is a high-quality TIFF-format public database created to provide a benchmark for algorithms that seek to detect retinal diseases in a multiclass problem. The database is labeled to distinguish between four disease stages—Normal, Choroidal Neovascularisation (CNV), Diabetic Macula Edema (DME) and Drusen [65].

#### 4.1.17. SERI DB

This 32-volume spectral domain OCT image database was created by the Singapore Eye Research Institute for Diabetic Macula Edema (DME) classification. It has equal volumes (16 each) for the DME and normal classes. Each volume comprises 128 B-scans with a resolution of 1024 × 512 pixels [66].

#### 4.1.18. ODIR Dataset

Ocular Disease Intelligence Recognition (ODIR) is a structured dataset of 5000 patients from the Peking University National Institute of Health Sciences. It consists of multiple label annotations for eight retinal diseases, namely Diabetes, glaucoma, Cataract, AMD, Hypertension, Myopi, normal and others. The images are saved in different sizes in the JPEG format. The distribution of the image class labels are as follows [67]: Normal: 3098 Diabetes: 1406 Glaucoma: 224 Cataract: 265 AMD: 293 Hypertension: 107 Pathological Myopia: 242 Other diseases: 791 Expert ophthalmologists were involved in the annotation exercise.

#### 4.1.19. OIA-ODIR

Shanggong Medical Technology Co. Ltd. assembled a real-life dataset of 5000 subjects in China [67]. The dataset is a large-scale multilabel disease detection database with 10,000 images captured by different cameras from different hospitals and regions of China. The images have various sizes [68].

#### 4.1.20. ROC (Retinopathy Online Challenge)

ROC has three different image types with varying resolutions because the photos were taken by various camera systems in different settings. The images were split into a training set and a test set, each with 50 images [69].

#### 4.1.21. IOSTAR

IOSTAR has 30 retinal photos taken by a laser fundus camera and edited by the same two specialists who were involved with the annotation of the DRIVE database. Optic disc and the A/V ratio annotations are also included in the IOSTAR database. The images have a resolution of 1024 × 1024 pixels [70].

#### 4.1.22. REVIEW (Retinal Vessel Image Set for Estimation of Widths)

Al-Diri in [71] describes REVIEW as a compound directory of four datasets of resolution higher than the DRIVE dataset. The four datasets are: High-Resolution Image Set, Vascular Disease Image Set, Central Light Reflex Image Set and the Kick Point Image Set. This database was designed for blood vessel segmentation model evaluation. It contains 16 images where 193 vessel segments showing an assortment of blood vessel types and pathologies were manually identified. There are 5066 manually marked profiles in this image set [48].

#### 4.1.23. DR HAGIS Database

This database was created as part of a DR screening campaign in the United Kingdom (UK). Images have different resolutions as they were taken at different centers using different fundus cameras. The database provides a gold standard on which the performances of DR algorithms can be compared [72].

#### 4.1.24. VAMPIRE (Vascular Assessment and Measurement Platform for Images of the Retina)

This database is a culmination of international corporations between five clinical centers and four image-processing research groups. VAMPIRE was captured by a fundus camera and software designed for the identification of retinal vessels.

#### 4.1.25. KAGGLE Database

The KAGGLE database, provided by EyePACS, contains 88,702 high-quality images taken by different cameras in different conditions. Of these, 35,126 form the training set, while 53,576 form the test set. Some KAGGLE images do contain noise (do have dots, circles, squares, etc.). DR detection algorithms should still be able to detect DR even under noisy conditions [73].

#### 4.1.26. RET-TORT

RET-TORT is a public database that contains 60 retinal images from patients with hypertension and healthy patients, including information relating to their estimated tortuosity. More details on RET-TORT are obtainable in [74].

### 4.2. Private Databases

Researchers have also used private databases to evaluate algorithm performance in the retinal disease detection space. To preserve the privacy of subjects and adhere to ethical provisions, the images are anonymized before they are used for model design and performance evaluation. In some cases, some private databases can be availed upon request to the authors and medical establishments who own the data or sponsored the research. We discuss, in this section, a few private datasets that have been used in this space.

#### 4.2.1. The RetCam3 Dataset

This is a private database created as a by-product of a premature infant screening program. A total of 80 images of resolution 640 × 480 pixels were captured by a RetCam3 camera [75].

#### 4.2.2. SCES

The Singapore Chinese Eye Study Ophthalmology (SCES) dataset was created in a screening study and has 1676 images, with 1630 normal and 46 glaucomatous images. This private database has images with resolutions of 3888 × 2592 and 3504 × 2336 pixels [76].

#### 4.2.3. TROPIC (Telemedicine for ROP in Calgary)

TROPIC contains retinal images taken from 41 premature infants. There are 130 images in total taken from a RetCam130 wide-angle camera with a resolution of 640 × 480 pixels. Of the 130 images, 110 were subsequently picked at random. A total of 30 images had no diagnosis, 30 had first-degree retinopathy of prematurity (ROP), 30 had second-degree ROP and 20 had third-degree ROP [77].

Table 2 is a summary of the retinal image databases discussed above, providing the number of images, resolutions, camera used and the purpose for which the databases were created.

### 4.3. Summary

This section presented the public and private retinal databases commonly used for the detection and grading of retinal diseases. The STARE and the DRIVE public databases are two of the most widely used retinal databases, owing to the superior resolutions of their images [49]. Privately owned databases can be accessed upon request to the owners. Algorithms can be trained from scratch, from pre-trained networks or on ensembles of CNN networks. Model performance depends on the number of images, pre-processing tasks, image quality and on the task at hand [79].

## 5. Review of Retinal Disease Detection Research

A critical review of the application of DL for classification of retinal pathologies is presented in this section. Case studies were drawn from diabetic retinopathy (DR), Age-Related Macular Degeneration (AMD), glaucoma and multiretinal disease applications. Segmentation methods were not part of this review.

### 5.1. Diabetic Retinopathy Classification

What makes DR the most important target for automatic detection is its persistence on the leaderboard of sight-threatening diseases among working-age adults. In [55], authors proposed IDx-DR X2.1, a DL device based on AlexNet, to detect DR severity. The purpose was to compare the performance of this device against a previously designed non-DL-based method called the Iowa detection Program (IDP). The authors used five DR levels; moderate, severe, non-proliferative DR, proliferative diabetic retinopathy (PDR) and/or Macular Edema (ME). The DL-based method outperformed the non-DL-based method. The model did not miss any cases of severe NPDR or ME. Specificity was higher than the specificity of IDP. The advantage of this study is that it was evaluated on a publicly available database. This has a positive bearing on the reproducibility of the method. The limitation of this method is on the dataset used, Messidor-2, which contains high-quality images not typical of those obtained in a clinical screening setup, and besides, the dataset only contains only one image per eye. This limits the area of the retina coverage. In [80], a four-class DR classification model was trained on 70,000 labeled retinal images to detect 0 (no DR), 1 (mild DR), 2 (moderate NPDR), 3 (severe NPDR) and 4 (PDR). Each patient was represented by two images, one for each eye. The model was evaluated on 10,000 fundus images from the Kaggle DR detection challenge dataset. This model outperformed state-of-the-art models. A significant contribution was the inclusion of images from both eyes, which meant a larger area of the retina was covered. In [81], entropy images were used in place of fundus photos, and they demonstrated that feature maps were produced more efficiently. A model proposed in [82] assists with explainability by incorporating heatmaps which highlight areas of lesion concentration. They utilized heatmaps to indicate the pixels in the image were involved in predictions at the level of the image. Apart from DR classification, this model detects lesions as well. The two-task method outperformed both other lesion-detection methods and other heatmap-generating algorithms for ConvNets. This method could be used to discover new biomarkers in image data, owing to its non-reliance on manual segmentation for the detection of relevant lesions. The model makes an attempt to address the lack of CNN model interpretability, which leads to a lack of trust with patients and clinicians. One important feature of this technique was that it managed to detect, with great precision, lesions in blurry images captured by hand-held retinography. This provides hope for DR screening with lower resolution images taken using cellular phones, making CAD of DR more accessible to poorer communities. One limitation of this method is the inferior database ground truth of 1 grade per image. This leaves room for grader subjectivity.

Two deep CNNs, Combined Kernels with Multiple losses Network (CKMLNet) and VGGNet with extra kernels (VNXK) were developed by [83]. The two networks are improvements of GoogleNet and VGGNet, respectively. They also introduced a color space, LGI, for DR grading via CNNs. The improved networks were evaluated on the Messidor and EyePac datasets and the best ROC performances of 0.891 and 0.887 were achieved for the CKMLNet/LGI and the VNXK/LGI networks, respectively. These performances compared well with those of the state-of-the-art methods in [84,85,86]. A five-class classification model to detect and grade DR into categories ranging from 0 to 4, 0 being no DR and 4 being proliferative DR was proposed in [87]. Authors used transfer learning on VGG-16 and VGG-19 and evaluated their method on the EyePacs database. The best performance they achieved was accuracy of 0.820, a sensitivity of 0.800 and a specificity of 0.8200. Classes 3 and 4 performed poorly owing to class imbalances not favoring them. The augmentation approach adopted by the authors could have caused this poor performance. Prior to augmentation, they grouped classes 1 to 4 and labeled this class 1 and the no DR class as class 0. They then proceeded with augmentation on the new classes and, in the process, missed correcting the limited counts for classes 3 and 4.

EfficientNet-B3 was employed as the backbone model by [88] to develop DR detection models on the APTOS dataset of 38,788 annotated images. The model obtained a Kappa score of 0.935 on the test set, and the authors concluded that their method performed at the level of experts. A major advantage and contribution of this work was the provision of a more structured way of uniformly scaling the three dimensions of the EfficientNet network—width, depth and resolution. This was an improvement from the arbitrary scaling of the same by other authors. The drawback of this method is its complexity, and besides, the evaluation metric that the authors used (Kappa) is a departure from the ones employed by most models (accuracy, sensitivity, specificity), making it difficult to compare the performance with other models.

Authors in [89] used the DenseNet-121 model to design a DR detection method and evaluated it on the same database as in [88], APTOS. Their research achieved good performance with an accuracy of 0.949, a sensitivity of 0.926 and a specificity of 0.971. A weighted Kappa measure of 0.88 was achieved for this model, a performance inferior to the EfficientNet model in [88] on the same dataset. The authors claimed their method had higher efficacy compared to some state-of-the-art models, which they did not mention, and besides, the basis of comparison where different datasets were used for evaluation may not be justifiable. This makes it hard to believe the authors’ conclusions. Jang et al. in [90] developed a DR classification system using a CNN model built on the Caffe framework and evaluated it using the Kaggle database, achieving accuracy of 0.757 on the binary classification problem (DR, no DR). The authors concluded that their model can be used for DR screening programs for large DR populations. The researchers, however, used only accuracy as their evaluation metric and claimed their model performs comparably with Pratt et al. in [91], who quoted performance accuracy alongside specificity and sensitivity as evaluation metrics. Their claim is unjustifiable because accuracy alone results in misleading outcomes in highly imbalanced datasets like the one they used for evaluation. Furthermore, the authors reduced the DR classification problem to a binary classification problem, which is a departure from the typical five-class classification problem as stipulated in the International DR Disease Severity scale [92,93].

A two-stage Deep CNN for lesion detection and grading DR severity was proposed in [94]. This multiclass model detected microaneurysms, hemorrhage and exudates with recall values of 0.7029, 0.8426 and 0.9079 with a maximum area under the curve value of 0.9590. A DR analysis method based on two-stage deep CNNs was proposed by [94]. The model was evaluated on a re-annotated Kaggle fundus image dataset and obtained a maximum accuracy of 0.973, specificity of 0.898 and sensitivity of 0.987. Whilst this model performed fairly well, it was designed to detect a limited number of lesions, and it would be useful to observe its performance on an expanded range of lesions. AttenNet, a multiclass deep Attention-Based Retinal Disease Classifier using the Densenet-169 as its backbone, was developed by [95]. It pays attention to critical areas that contain abnormalities, a feature that helps visualize the lesions and possibly helps interpret the outcomes of the model. AttenNet achieved a four-class accuracy of 97.4%, a binary class sensitivity of 100%, with a specificity of 100%. The major contribution of this work was its high performance and an attempt to provide model explainability. Its limitation, though, is its potential computational expense owing to the complexity of the DenseNet-169.

Using the Kaggle dataset of 35,126 color fundus images, authors in [6] proposed a DL ensemble for predicting the five DR classes; normal, mild, moderate, severe and PDR. They used a collection of five CNN architectures: Resnet50, Inception V3, Xception, Dense 121 and Dense 169. The authors claimed that the model detected all DR stages and performed better than state-of-the-art methods on the same Kaggle dataset and yet, evidently, with a sensitivity of 0.515, specificity of 0.867 and accuracy of 0.808, this method trails behind a few models, such as the DCNN in [94] and the CKML in [83], evaluated on the same dataset. Jiang et al. in [96] presented an explainable ensemble DL model for DR disease classification. They integrated several deep learning algorithms (Inception V3, Resnet152 and Inception-Resnet-V2) and used the Adaboost algorithm to minimize bias in each individual model. The work provides weighted class activation maps (CAMs) to explain the results of the DR detection. CAMs illustrate the suspected position of the lesions. This research performed better than single deep learning models, producing an AUC of 0.946 for the integrated model against an AUC of 0.943 for the best-performing individual model. The Adaboost algorithm helped the models reach a global minimum. Prior to model development, the images underwent augmentation to increase their diversity. The dataset they used is private, and this poses potential accessibility challenges in the event of the need to confirm their results. In [97], the authors proposed ensemble classification methods combined with vessel segmentation for the detection of diabetic retinopathy. While the paper proposes an innovative and promising method for retinal disease prediction using deep learning techniques, the authors did not provide more detail on the datasets used for testing the proposed method, as well as the performance metrics used to evaluate its effectiveness. This makes it difficult to evaluate the proposed methods against other methods in the literature. The paper provides a comprehensive overview of the method used; however, the deep learning methods used in the ensemble were not mentioned, making it difficult for readers to understand how the models were combined and how each model affected the final performance metrics. A novel method that combines a Deep Convolutional Neural Network and vessel segmentation was presented in [98] for the early detection of proliferative diabetic retinopathy. The proposed method achieved an area under the curve (AUC) performance of 0.969, an accuracy of 94.1%, a specificity of 95.7% and a sensitivity of 92.7% on the MESSIDOR-2 database. These performances mean the proposed method can effectively distinguish between a diseased retina and a non-diseased retina. The small size of the dataset, lack of interpretability analysis and the fact that authors did not make an attempt to compare their method against other segmentation methods serve as the limitations to the proposed method. It would be hard to believe this method is generalizable, and besides, clinicians may find it hard to entrust patients’ lives on a black box method whose decision-making process remains opaque.

In [99], ViT-DR, a vision transformer-based model for DR detection on fundus images, is presented. The model was evaluated on four publicly available datasets: MESSIODOR-2, e-ophtha, APTOS and IDRiD. AUC scores of 0.956, 0.975, 0.946 and 0.924 were obtained for the datasets, respectively. The authors provide a detailed analysis of the model’s attention maps, which highlights the areas of the fundus images that the model is focusing on during the classification process. This way, users will have an idea of how decisions are made. The model is a promising approach for diabetic retinopathy grading using fundus images, but further research is needed to evaluate its generalizability to other tasks and its computational efficiency. A lesion-aware vision transformer network was proposed for DR detection in [100]. The authors’ approach leverages lesion awareness to improve the model’s performance in detecting and grading diabetic retinopathy. The model was evaluated on the MESSIDOR-2, e-ophtha and APTOS databases, achieving AUC scores of 0.956, 0.977 and 0.947, respectively. The performance of this network was quite comparable to the ViT proposed in [99], including the provision for model explainability. This model’s effectiveness for the detection of different types of lesions in clinical settings is yet to be established. A vision transformer that incorporates a residual module was presented in [101] for the classification of DR severity. The model achieved an accuracy of 0.893 on the MESSIDOR-2 dataset and an AUC of 0.981 on the APTOS dataset. The inconsistency in reporting performances, for example, the absence of AUC score for the MESSIDOR-2 dataset and accuracy for the APTOS dataset, is concerning. It is not possible to draw comparisons with other models, and besides, the performance of this model on these datasets is not fully specified. The authors have not provided an interpretability analysis for this model. Therefore, it remains difficult to appreciate how classification decisions are made. The authors of [102] developed an ensemble of transformer-based models coupled with attention maps for the detection of DR. The model was evaluated on the MESSIDOR-2 and the APTOS datasets and achieved AUC scores of 0.977 on MESSIDOR-2 and an accuracy of 0.912 on the APTOS dataset. A major contribution of this work was the improvement in performance and the inclusion of the attention module to help clinicians understand the underlying pathology better. Critical omissions from this work include the lack of analysis of its performance against other models and also computational efficiency comparisons against CNN-based models. These are important aspects in considering the clinical applications of a model.

Table 3 is a summary of the DL-based models applied to detect diabetic retinopathy.

#### 5.1.1. Discussion

The studies reviewed in this section have shown that DL techniques outperform traditional methods in diagnosing and classifying DR. For example, in [55], the authors developed a deep learning device, IDx-DR X2.1, which outperformed the Iowa Detection Program (IDP), a non-deep learning-based method. The model achieved high sensitivity and specificity and did not miss any cases of severe NPDR or macular edema. Similarly, authors in [80] developed a four-class DR classification model that outperformed state-of-the-art models. The authors also included images from both eyes, allowing for a more extensive coverage of the retinal area. The MESSIDOR-2 and EyePacs databases were the most commonly used databases in the papers reviewed in this work.

One of the most significant contributions of the reviewed studies is the use of DL models for lesion detection and grading of DR severity. For instance, [94] developed a two-stage deep CNN for lesion detection and grading DR severity, while [82] proposed a model that assists with the explainability by incorporating heatmaps in the model. These models demonstrated the potential of deep learning techniques in detecting DR lesions, which can be a useful assistive tool in clinical practice, especially if it has explainability embedded in it.

Another advantage of the deep learning models developed in the reviewed studies is their potential to be used in resource-limited settings, such as developing countries. For example, in [90], authors developed a DR classification system using a CNN model built on the Caffe framework and evaluated it using the Kaggle database. They achieved high accuracy on the binary classification problem (DR, no DR), demonstrating the potential of deep learning in providing an accessible tool for DR screening programs for large DR populations.

However, there are some limitations to the studies reviewed. One of the limitations is the small dataset size used in some studies, which may pose generalizability challenges. Another limitation is the lack of interpretability of some deep learning models, which may hinder their acceptance and use in clinical practice. The evaluation metrics used in some studies were also limited, and this may affect the generalizability of the models developed.

#### 5.1.2. Summary

This review explores recent advances in the use of DL methods to detect and diagnose diabetic retinopathy (DR). The authors examined several studies that classify DR into different categories, ranging from no DR to proliferative DR, and evaluated the strengths and limitations of each approach. Some of the most promising methods use ensemble models or innovative techniques, such as entropy images or lesion detection.

One of the biggest challenges faced by researchers in this field is the lack of standardized datasets and ground-truth annotations for DR. Many studies use publicly available datasets, which may not be representative of real-world screening situations. Additionally, some studies rely on limited or imbalanced datasets, which may lead to biased results.

Overall, the authors conclude that deep learning methods show great promise for improving DR screening and diagnosis. However, further research is needed to address issues such as dataset bias and lack of interpretability and to determine whether these methods can be applied effectively across different populations and screening settings.

### 5.2. Age-Related Macular Degeneration Classification

Some recent results on AMD classification using convolutional neural networks are presented in this section. The outcomes of the preliminary work were presented in [103]. They applied transfer learning to fine-tune a DCNN for the purpose of detecting individuals with intermediate-stage AMD. Accuracies up to 0.950, sensitivities of 0.964 and specificities of 0.956 with no hyperparameter fine-tuning were attained on the AREDS dataset. Higher performances would probably have been recorded with fine-tuning and with a bigger training dataset. The model proposed in [104] performed binary classification between early-stage AMD and advanced-stage AMD using Deep CNN on the AREDS database. This model was compared with earlier models that combined deep features and transfer learning. The researchers concluded that applying deep learning-based methods for AMD detection leads to results similar to human experts’ performance levels. A deep CNN-based method with transfer learning to assist in identifying persons at risk of AMD was proposed in [79]. This model was evaluated using the AREDS database with 150,000 images. They used an enhanced VGG16 architecture employing batch normalization. The authors solved a binary and a four-class problem, achieving between 83% and 92%. As their main contribution, the authors debunked the belief that transfer learning always outperforms networks trained from scratch. Their network, trained from scratch with sufficient images, produced higher accuracies compared to accuracies obtained using transfer learning. Network depth has a positive bearing on performance, as observed with VGGNet-16 outperforming shallower networks, such as AlexNet, on similar tasks. The work of [105] involved the development of an AlexNet model for classifying OCT images into healthy, dry AMD, wet AMD and DME types. The method trains the network from scratch without using transfer learning. It was evaluated on a four-class problem and two, binary class combinations. The method performed better than that of presented in [18], who used transfer learning and evaluated their network on the same dataset. The advantage of this network is the high number of training images (83,484). What makes these results important is that AlexNet is less computationally expensive compared to its successors, and yet it is achieving some performance improvements. The marginal performance improvement in this method though, compared to the model by Kermany et al. in [18] may not justify foregoing the computational efficiencies afforded by transfer learning.

In [106], a 14-layer deep CNN was evaluated using the blindfold and cross-validation strategies on some private AMD retinal database, resulting in accuracies as high as 95.17%. Three fully connected layers, four max-pooling layers and seven convolutional layers were implemented in this work. Adam optimizer was employed in parameter tuning. Matsube et al. in [107], designed a network with three convolutional layers with ReLU unit and max-pooling layers and evaluated it on pre-processed fundus images. The Deep CNN fared well against human grading by six ophthalmologists. The authors deemed their system capable of identifying exudative AMD with high efficacy and useful for AMD screening and telemedicine. An ensemble of several CNN networks was proposed in [108] to classify among 13 different AMD classes on the AREDS database. The model outperformed human graders on the AREDS database, and they deemed it suitable for AMD classification in other datasets for individuals with ages 55 years and above. Authors in [109] sought to analyze the impact of image denoising, resizing and cropping for AMD detection. The authors observed that a reduction in image size would not lead to a significant reduction in performance, and yet results in a substantial reduction in the model size. They also concluded that the model’s highest accuracies were obtained with original images, without denoising and cropping. AMDOCT-Net fared better than VGG16 and OCT-Net architectures for comparable model sizes. This work produces significant results regarding image resizing; it significantly reduces model size with an insignificant reduction in performance. The authors of [110] proposed a vision transformer network for AMD classification and detection. They evaluated the model on the MESSIDOR and the APTOS databases, achieving an accuracy of 0.913 with APTOS and an AUC score of 0.963 on the MESSIDOR dataset. The major contributions of this work include the high performance of the model and the explainability capability inherent with vision transformers. The limitation of this model is that the attention maps may not always align with the underlying pathology, which could lead to incorrect diagnoses. In [111], a vision transformer network was proposed for AMD diagnosis on retinal fundus images and was evaluated on the AREDS dataset. The model achieved an accuracy of 0.994 on the four-class classification task and an AUC of 0.993 on the binary classification task. As a contribution, this work shows that AMD detection assistive tools can be developed using ViTs and achieve performances comparable to state-of-the-art CNN models but with the added advantage of explainability to enhance trust with clinicians and patients alike. The drawback of this model, though, is that it was not evaluated on many AMD datasets to allow for generalizability.

#### 5.2.1. Discussion

This section reviewed several studies that applied DL methods for the classification of Age-Related Macular Degeneration (AMD). A plethora of studies have demonstrated great potential in the use of DL methods for the classification of AMD stages and also to differentiate between healthy and AMD-affected eyes. Most studies reviewed evaluated their models on the AREDS database.

Transfer learning has been applied in a lot of the studies, examples of which are [103,104], to fine-tune pre-trained DL network architectures for the classification of AMD. The results show accuracies of up to 0.950, sensitivities of 0.964 and specificities of 0.956, which compare closely with the performance levels of human experts. It was, however, observed in [79] that a network trained from scratch with sufficient input images could produce higher accuracies compared to models fine-tuned on pre-trained models.

The study observed that the depth of the network also impacts model performance. This was demonstrated by a VGGNet-16 network outperforming shallower networks, such as AlexNet, for similar tasks. AlexNet was utilized in [105] for the classification of OCT images into healthy, dry AMD, wet AMD, and DME types without using transfer learning. The high number of training images (83,484) used in this study contributed to its better performance compared to transfer learning-based methods.

Other studies have investigated the impact of denoising, resizing and cropping images on the accuracy of AMD detection. Studies by [109] showed that reducing the image size does not significantly reduce performance, and yet results in a substantial reduction in the model size’s computational expense. They also concluded that the highest accuracies were obtained with original images, without denoising and cropping. In [110,111], vision transformers were employed for AMD classification, achieving high accuracy and AUC scores on the MESSIDOR, APTOS and AREDS databases. The major contribution of these papers is the explainability capability inherent in the ViT models, which enhances trust with clinicians and patients alike.

Overall, the papers reviewed show that deep learning-based methods, including both CNNs and ViTs, have the potential to achieve performance levels similar to human experts in AMD classification. However, limitations of the models include a lack of generalizability and the potential for incorrect diagnoses due to attention maps not aligning with the underlying pathology. Additionally, it is important to carefully consider the trade-offs between transfer learning and training from scratch when developing AMD classification models.

#### 5.2.2. Summary

This section discussed recent developments in using deep learning models, specifically CNNs and vision transformers, for Age-Related Macular Degeneration (AMD) classification. Several studies have shown promising results in using these models to classify retinal fundus images for various stages of AMD, with some achieving high levels of accuracy and outperforming human graders. The use of transfer learning and network depth has also been explored, with some studies showing that training networks from scratch with sufficient data can produce higher accuracies compared to using pre-trained models. However, there is still room for improvement, particularly in terms of generalizability to different datasets and addressing potential limitations of the models, such as the alignment of attention maps with underlying pathology in vision transformers.

Table 4 summarizes the main algorithms for AMD detection.

### 5.3. Glaucoma

An early work in glaucoma detection was presented in [112]. The authors proposed a CNN employing dropout and data augmentation to improve convergence. The CNN network had six layers, four convolutional layers of decreasing filter sizes and two dense (FC) layers. The model was evaluated on the ORIGA and SCES datasets and achieved an AUC measure of 0.831 on the ORIGA database and 0.887 on the SCES database. Neither the specificity nor the sensitivity of this network was reported, raising doubts about whether this network did not suffer from overfitting, which is typical with imbalanced data in such domains. The Inception-V3 pre-trained architecture was designed in [23] to predict glaucomatous optic neuropathy (GON). The images were first graded by expert ophthalmologists, and the local space average color subtraction technique was employed to accommodate for varying illumination. The authors claimed the model was capable of detecting referable GON with high sensitivity and specificity. False positive and false negative results were caused by the presence of other eye conditions. In [113], the researchers took advantage of domain knowledge and designed a multibranch neural network (MB-NN) with methods to automatically extract important parts of images and obtain domain knowledge features. The model was evaluated on datasets obtained from various hospitals and achieved an accuracy of 0.9151, a sensitivity of 0.9233 and a specificity of 0.9090. ResNet-50 was used as a base network to implement a deep CNN for the detection of early glaucoma. A proprietary database with 78 images was used to train the model, and 3 additional public datasets were used to validate it. A validation accuracy of 0.9695 was achieved. Whilst most methods focus on advanced glaucoma detection, this method’s focus is early detection, a more difficult and important task of detecting the more subtle changes to the images. The few training images made the model more susceptible to overfitting. The DenseNet-201 network in [114] was developed as a model for the detection of glaucoma. The model was evaluated on the ACRIMA dataset and obtained a maximum accuracy of 0.97, F1 score of 0.969, AUC of 0.971, sensitivity of 0.941 and specificity of 1.0. This model performed better than the authors’ previous work in [115] where they experimented with ResNet-121. An added advantage of the DenseNet network is its ability to manage the diminishing gradient problem. DenseNet suffers from computational inefficiency owing to its deep layers and millions of parameters.

An attention-based CNN network for glaucoma detection (AG-CNN) was proposed by [64]. The network was trained on an 11,760-image LAG dataset. Attention maps were used to highlight salient regions of glaucoma. The model performed better than state-of-the-art networks on the same database and also on RIM-ONE public database. The best performances were accuracy: 96.2%; Sensitivity: 95.4%; Specificity: 96.7% and AUC: 0.983. The main contribution and advantage of this paper was the introduction of visualized heatmaps that helped to locate small pathological areas better than the other methods. This helps with model explainability. The limitation of their network is that it adds more weight parameters to the model, increasing the computational complexity. The authors of [116] proposed a deep learning method for glaucoma detection that combines optic disc segmentation and transfer learning. The model, which was fine-tuned on a pre-trained ResNet50 model, was evaluated on two publicly available image databases, DRISHTI-GS1 and RIM-ONE V3, achieving accuracies of 98.7% and 96.1%, respectively. A significant contribution of the authors was an analysis of model interpretability. Whilst good performances were recorded with this method, the small sizes of the datasets and the limited number of datasets on which the model was evaluated adversely affect its generalizability. Moreover, it would have been easier to compare the performances of this model with other segmentation models in the literature had the authors had a wider range of evaluation metrics, such as specificity, sensitivity and F1 score. In the work [117], a vision transformer for glaucoma detection was proposed and evaluated on the ORIGA and RIM-ONE v3 datasets, achieving a sensitivity of 0.941 and a specificity of 0.957 on the RIM-ONE v3 dataset and a sensitivity of 0.923 and a specificity of 0.912 on the ORIGA dataset. The paper provides a thorough analysis of the model’s attention maps, which can help clinicians understand the underlying features that contribute to the model’s decision-making process. Additionally, the authors did compare the performance of their model with state-of-the-art models, providing an opportunity for readers to judge the strengths and weaknesses of different models. The small size of the datasets used for evaluation makes it hard to generalize the performance of their approach. There is a need for additional validation with larger and more diverse datasets. In the work of [118], the ORIGA dataset was used to evaluate a ViT model for glaucoma classification. An AUC of 0.960 for binary classification and an F1 score of 0.837 for multiclass classification were registered. The authors managed interpretability well by providing a detailed analysis of the model’s attention maps, which help identify important features associated with glaucoma. However, like in [117,119,120], readers will be skeptical about generalizing the performance of the model owing to the small size of the ORIGA, RIGA and RIM-ONE v3 datasets used for evaluation. In the work of Seremer et al. [121] transfer learning was applied to train and fine-tune the ResNet-50 and the GoogleNet networks for early and advanced glaucoma classification. The models were evaluated on the RIM-ONE public dataset. It was observed that the sensitivity values were very low for both GoogleNet and ResNet, reaching as low as 0.17. Specificities as high as 0.98 were achieved with the GoogleNet architectures for early glaucoma detection. GoogleNet was also reported to have shorter execution times compared to ResNet. A multistage DL model for glaucoma detection based on a curriculum learning strategy was proposed in [122]. The model included segmentation of the optic disc and cup, prediction of morphometric features and classification of the disease level (healthy, suspicious and glaucoma). The model performed better than state-of-the-art models on the RIM-ONE-v1 and DRISHTI-GS1 datasets, with an accuracy of 89.4% and AUC of 0.82. Omitting specificity and sensitivity of the model raises questions about possible overfitting owing to imbalanced data. The performances of DL techniques for the detection of glaucoma are summarized in Table 5.

#### 5.3.1. Discussion

Glaucoma is a leading cause of blindness, and deep learning (DL) techniques have been employed to aid its detection. Several studies have proposed various DL models that employ different architectures, including Inception-V3, ResNet-50, DenseNet-201 and vision transformers, for detecting glaucoma. Attention-based CNN networks, transfer learning and multistage DL models have also been proposed. Most studies focus on detecting advanced glaucoma, but some focus on early detection, which is more challenging. While these models, most of which were evaluated on the RIM-ONE v3 database, achieved high accuracy, sensitivity and specificity on their respective datasets, they have limitations, such as small dataset size, limited diversity and limited evaluation metrics. Thus, additional validation with more diverse and larger datasets is needed to generalize their findings better. Additionally, there is a need for interpretability and model explainability. Overall, the performance of DL techniques for glaucoma detection is promising, and they have the potential to improve the accuracy and efficiency of glaucoma diagnosis.

#### 5.3.2. Summary

Several deep learning models have been proposed for glaucoma detection using various techniques, such as CNNs, attention-based networks, transfer learning and curriculum learning. These models were evaluated on different datasets and achieved good accuracy, sensitivity and specificity measures. However, the small size and limited number of datasets used for evaluation affect their generalizability. The visualized heatmaps introduced in some models aid in locating small pathological areas, while others focus on early detection, a more challenging task. The choice of architecture and evaluation metrics depends on the specific requirements of the detection task.

### 5.4. Multiple Retinal Disease Detection

This section presents a review of studies that targeted classifying between AMD, DR, glaucoma and other retinal diseases in multiclass tasks or in multiclass, multilevel tasks. Using EfficientNet-B3 as the base model, authors in [67] developed a DL model merged with a mixture loss function for automatic classification between glaucoma, cataract and AMD in a four-class problem, including normal. The mixture loss function was a hybridization of the focal loss and the correntropy-induced loss functions combined to minimize the effects of outliers and class imbalance. The 5000-image OIA-ODIR dataset was used for model evaluation. The FCL-EfficientNet-B3 model outperformed other baseline methods for the detection of the three retinal diseases. The main advantages of their model include the reduction of computation cost and training speeds. EfficientNet scales well, but it is hard to achieve a balance in its three dimensions. The model also struggled to correctly classify AMD and glaucoma. An ensemble of three ResNet-152 networks was proposed in [123] for classifying Choroidal Neovascularization (CNV), Diabetic Macula Edema (DME), Drusen and normal. The ensemble method outperformed a single ResNet-152 network, posting a maximum accuracy of 0.989, sensitivity of 0.989 and specificity of 0.996. The authors carried out experiments with different size datasets and concluded that model performance improved with more training data. The model has a drawback of the increased computational complexity owing to the large number of layers and parameters in ResNet-152.

Kamran et al. in [124] proposed an architecture to differentiate between a range of pathologies causing retinal degeneration. The authors claim their model outperforms expert ophthalmologists. In [125], an ensemble, four-class classification model to automatically detect Choroidal Neovascularization (CNV), Diabetic Macula Edema (DME), Drusen and normal in OCT images based on the ResNet50 neural network was presented. This model, which the authors claim performs better than ophthalmologists with significant clinical experience, attained an accuracy of 0.973, a sensitivity of 0.963 and a specificity of 0.985. Global accuracies of up to 0.95 were attained in [126] with their deep learning classifier of inherited retinal diseases using fundus autofluorescence (FAF). Their classifier detected retinitis pigmentosa, stargardt disease and normal out of 389 images. A CNN-automated multiclass classifier for retinal diseases using spectral-domain OCT images was developed by [3]. The model detected AMD, Choroidal Neovascularization (CNV), Diabetic Macula Edema (DME), Drusen and normal cases. The model correctly detected AMD with 100% accuracy, CNV with 98.86% accuracy, DME with 99.17% accuracy, Drusen with 98.97% accuracy and normal with 99.15% accuracy. The overall accuracy achieved was 95.30%. Gour and Khanna (2020) proposed an automated multiclass, multilabel transfer learning-based CNN for the detection of ocular diseases. Leveraging the power of transfer learning, they built two models using four CNN architectures, VGG16, InceptionV3, MobileNet and ResNet and evaluated the models on the ODIR database to predict the presence or absence of eight ocular diseases from the dataset. Model 1 passes the left and right eye images separately as inputs to the CNN architectures for feature extraction before the features are later concatenated. Model 2 concatenates the images followed by feature extraction. For both models, the architectures were trained for 100 epochs and the sigmoid activation function was used to predict the probability of each of the eight labels corresponding to the eight ocular diseases depicted in the ODIR database. The disease categories represented in the database are normal (N), Diabetes (D), glaucoma (G), Cataract (C), AMD (A), Hypertension (H), Myopia (M) and other diseases (O). The VGG16 architecture with SGD optimizer on model 1 outperformed the other architectures, achieving AUC and F1 score values of 84.93 and 85.57, respectively. This work provides a fairly viable solution to the multiclass, multilabel classification problem for the prediction of ocular diseases, but its limitation was the low performance of categories with fewer images owing to the imbalanced nature of the dataset. Table 6 presents a summary of the DL-based methods for the detection of multiple retinal diseases. An Ensemble Label Power-set Pruned datasets Joint Decomposition (ELPPJD) technique was developed in [127] to solve the multiclass, multilabel classification problem. They transformed the multilabel problem into a multiclass classification problem. They adopted 10-fold cross-validation and used average accuracy, precision, recall and F-measure to evaluate the models. The authors developed two variants of the ELPPJD method, ELPPJD_SB (size-balanced strategy) and ELPPJD_LS (Label similarity), two decomposition strategies in ELPPJD. ELPPJD_LS outperformed not only ELPPJD_SB but also two widely used multilabel classification methods, RAkEL and HOMER. ELPPJD_SL produced an average accuracy of 88.59%, a good result in multiclass classification [127]. The authors utilized transfer learning and fine-tuning techniques in [128] to adapt a pre-trained Inception-v3 architecture, combining it with a novel feature attention layer for the prediction of four common retinal diseases, diabetic retinopathy, Age-Related Macular Degeneration, glaucoma and retinal vein occlusion. With the feature attention layer helping to highlight important regions of the input image, the model had some remarkable accuracies, outperforming state-of-the-art models in the process. Specifically, EyeDeep-Net achieved an accuracy of 95.4% on the IDRiD dataset and an accuracy of 96.5% on the MESSIDOR dataset for multiclass classification. Whilst this method achieves considerably good accuracies compared to state-of-the-art methods, the datasets used were comparatively small, which may affect the generalizability of the model. Moreover, the authors did not provide a thorough interpretability analysis of the proposed method, which could have helped understand the model’s decision-making process. A vision transformer was presented in [129] for the classification of multiple diseases in fundus images. Evaluation performed on the IDRiD, Messidor-2 and APTOS datasets yielded promising accuracies of 0.9847, 0.9667 and 0.9576, respectively. The authors performed extensive experiments to evaluate their approach and provide a detailed analysis of the model’s attention maps to identify the regions of interest for each disease. Although the authors compared their results with those of previous studies on individual diseases, they did not compare their approach with other multidisease classification models. There was no attempt by the authors to provide an analysis of the computational cost of their model with CNNs, which have been dominating computer vision. A novel attention-guided approach to identify the most important regions in retinal images for disease classification was proposed by [130]. The authors demonstrated that their approach outperforms several state-of-the-art models on two publicly available datasets, achieving a macro F1-score of 0.871 on the MESSIDOR-2 dataset and 0.845 on the EYEPACS dataset. The use of attention-guided vision transformers, which can improve the interpretability of the model’s predictions and provide insight into the most important regions for disease classification, was a major contribution of their work. However, the authors failed to provide a discussion of the computational complexity of their model. Given the large number of parameters in vision transformer-based models, the computational cost of training and deploying the model may be a limiting factor in real-world clinical applications. Two deep learning architectures, RetinaNet and ViT, were combined in the work of [131] for the automated detection of retinal diseases. Their method achieved state-of-the-art performance on the IDRiD and the MESSIDOR-2 datasets, scoring a sensitivity of 0.944 and a specificity of 0.966 on the IDRiD dataset and an accuracy of 0.971 on the MESSIDOR-2 dataset. One limitation of this model is the lack of discussion on the explainability of the model. Given the black-box nature of deep learning models, it would be valuable to provide insights into the most important regions of the retinal images for disease detection. An approach for multilabel classification of retinal diseases using a self-attention mechanism-based Vision Transformer was proposed in [132]. The authors demonstrated that their approach outperforms several state-of-the-art models on the Kaggle Diabetic Retinopathy Detection (KDD) dataset, achieving a mean F1-score of 0.865 and an accuracy of 0.897. The use of a self-attention mechanism-based ViT allows the model to focus on relevant features in the retinal images for disease detection. However, one limitation of this paper is the lack of evaluation of other publicly available datasets, which limits the generalizability of the proposed approach. Additionally, the authors do not provide insights into the most important regions of the retinal images for disease detection, which limits the interpretability of the proposed approach.

#### 5.4.1. Discussion

The use of deep learning (DL) models for the detection and classification of retinal diseases is a promising area of research, with numerous studies showing significant progress in recent years. However, there are several critical issues that need to be addressed in order to improve the reliability and generalizability of these models.

One of the primary challenges is the lack of diverse and well-annotated datasets. Many studies have reported using relatively small datasets, and the lack of diversity in these datasets can limit the generalizability of the developed models. Moreover, it is important to consider that the prevalence of retinal diseases varies widely across different populations and ethnicities. This can limit the generalizability of models developed using datasets from a specific population or region. Therefore, efforts to collect and annotate large, diverse datasets are critical to ensure the generalizability of these models. The MESSIDOR-2 database was the most frequently used database for evaluating the models.

Another challenge is the interpretability of DL models. It is often difficult to understand how these models arrive at their predictions, which can limit their utility in clinical settings. While some studies have proposed the use of attention mechanisms or visualization techniques to identify important regions in retinal images, more research is needed to develop methods for interpreting the predictions of DL models.

Additionally, DL models require significant computational resources for training and inference, which can limit their scalability and feasibility in clinical settings. Therefore, there is a need for more research on developing efficient DL models that can be trained and deployed on resource-constrained devices.

Finally, it is important to recognize that DL models should not replace expert ophthalmologists. While these models can provide valuable insights and support to clinicians, they should be used as a tool for aiding diagnosis and not as a replacement for clinical expertise.

#### 5.4.2. Summary

This section presents an overview of several studies that have targeted the classification of multiple retinal diseases using deep learning (DL) models. Common approaches used in these studies are pre-trained convolutional neural networks (CNNs), such as ResNet, EfficientNet and ViT, and ensemble methods. The main challenges are class imbalance and the interpretability of DL models. Some studies have proposed the use of mixture loss functions or transfer learning to overcome class imbalance and attention mechanisms or visualization techniques to improve interpretability. The reviewed studies have shown promising results, but larger and more diverse annotated datasets are needed to improve generalizability, and more research is needed on the interpretability and explainability of DL models.

## 6. Discussion

While the DL approach to retinal disease detection brings with it a lot of positives, there are a number of challenges that still need to be overcome. This section discusses some of the challenges associated with the DL approach, including image acquisition challenges, model training challenges and the lack of explainability of DL approaches.

Convolutional Neural Networks (CNNs) and ViTs thrive on huge amounts of data for better performance, but retinal images are seldom found in big numbers and are usually not annotated. DL models tend to overfit when trained with little data. Data augmentation, transfer learning and generative adversarial neural networks (GANs) have been used to try and mitigate the overfitting challenge [133,134,135,136,137]. GANs are hard to train; the model parameters oscillate and do not converge easily [138]. Often, pre-trained networks used in transfer learning belong to a different domain, possibly adversely affecting the performance of such networks due to a lack of domain adaptation. These shortcomings make the overfitting problem an open research issue. There is a significant number of misdiagnosed abnormalities due to limited visibility of the lesions, low image contrast or noisy images. There have been quite some advances in this area, but choosing the right pre-processing techniques to achieve satisfactory CNN classification accuracy remains a problem worth pursuing. In medical imaging classification and segmentation problems, there is usually an imbalance between the positive and negative classes. This leads to bias in classification, where more common classes get favored. Because of this challenge, it is not sufficient to rely on accuracy alone as the ultimate measure of performance, sensitivity and specificity have often been used, in addition to accuracy, to provide a more realistic overall performance evaluation. There has not been enough research to establish the effect of imbalanced data on the performance of CNNs.

Training DL models is an iterative process that involves repetitively computing the derivative of the loss function, which, in turn, causes the vanishing gradient problem, especially when the sigmoid function is used as the activation function. The dying ReLU problem is a version of the vanishing gradient problem that is experienced when the ReLU activation function is used [139]. InceptionNet tried to mitigate the vanishing gradient limitation inherent in some CNN architectures, but this problem has not been sufficiently addressed [139,140,141,142]. Deep networks are associated with great performance, but they are computationally expensive. There is still an ongoing search for lightweight CNNs with sufficient generalization capabilities [91,143,144]. The choice of hyperparameters influences the performance of CNNs. A small change in the hyperparameter values can have a significant bearing on the overall performance of the CNN model. The design of hyperparameter optimization strategies is a research area worth pursuing [145]. Combining multiple and diverse architectures as network ensembles can help to improve the generalizability of diverse categories of images [146,147]. There have been successful research on the application of ensembles in other domains, but there has not been much research on the extent of performance of ensemble CNN architectures in multiple retinal disease detection.

Neural Networks, by their very nature, are black boxes and their outputs are not easily interpretable. This raises trust issues with patients and clinicians alike. Developers of DL methods are at pains explaining how their models arrived at a conclusion. There have been attempts to use class activation maps (CAM) to activate pixels in regions where the lesions exist, but sometimes CAM activates pixels far from the relevant object [148,149]. Model explainability, therefore, remains an area of active research, especially with CNNs. ViTs can be computationally complex and require large amounts of training data. This can limit their applicability in some scenarios. They have built-in interpretability features, such as self-attention mechanisms, that allow the model to focus on relevant features in the input image. This can make ViTs more suitable for building explainable models compared to traditional CNNs. Further research is needed to better understand the trade-offs between interpretability and computational complexity in ViTs and to identify the most effective techniques for building lightweight explainable models using ViTs [150].

More often than not, a single retinal image could contain more than one disease, therefore, there is a need for models capable of detecting multiple diseases from single images [14]. There is a number of challenges to achieving this feat. One of the challenges is the lack of large-scale, high-quality annotated datasets, which is critical for training deep learning models. Many existing datasets only contain a limited number of images with a single disease label, which makes it difficult to train deep learning models for multiple disease detection. Another challenge is the high variability and complexity of retinal diseases, which can lead to high false-positive or false-negative rates in disease detection. Retinal images can also contain various artifacts and noise, which can negatively impact the accuracy of disease detection [151,152].

The detection of retinal diseases using CNNs and ViTs faces an uncertainty problem due to the complex and variable nature of retinal disease manifestations and variability in image quality and other imaging artifacts [153]. This variability can make it challenging for deep learning models to accurately detect and classify retinal diseases, particularly when the training data are limited or does not fully capture the variability of the disease [154]. Additionally, retinal images can be subject to variability in image quality, lighting conditions and other imaging artifacts, which can further increase uncertainty in automatic detection. For example, the presence of imaging artifacts such as blurring or distortion can make it difficult for deep learning models to accurately detect disease features in the image. To address these challenges, various approaches have been proposed, including the use of large and diverse datasets, data augmentation techniques and the incorporation of contextual information into deep learning models. These approaches can help to reduce uncertainty and improve the accuracy and reliability of automatic detection of retinal diseases using CNNs and ViTs. However, ongoing refinement and validation of these approaches are necessary to ensure their effectiveness and reliability in clinical practice [137,150,155,156].

In the case when there is limited information available, such as limited training data or a lack of diversity in the dataset, the performance of deep learning models for retinal disease detection may be negatively impacted. This can be particularly challenging when trying to detect rare or complex diseases, where the limited information environment may make it difficult for the model to learn the necessary features to accurately classify the disease [151]. Transfer learning and data augmentation techniques can be effective solutions to improve the models’ performance. However, further research is needed to explore the optimal combination of these techniques and to evaluate their effectiveness in clinical practice [157,158,159].

In the case where there is limited time and a small number of data available, the development of deep learning models for retinal disease detection using CNNs and ViTs can be challenging. The limited amount of data may not be sufficient to train a deep learning model, and this can lead to overfitting or poor performance on unseen data. Additionally, the limited time available for model development and optimization can result in suboptimal performance [154,160,161,162]. To address these challenges, researchers have explored various approaches, such as transfer learning, data augmentation and the use of smaller, more focused datasets. These approaches can help to improve the performance of deep learning models even in a limited data environment. For example, transfer learning can be used to leverage pre-trained models to learn new features and improve performance on limited data, while data augmentation can be used to artificially expand the dataset and improve model generalization. Recent studies have shown promising results for the use of these approaches in retinal disease detection using both CNNs and ViTs. However, further research is needed to fully evaluate their effectiveness in clinical practice, particularly for rare or complex retinal diseases where limited data are available. Overall, the development of deep learning models in a limited data and time environment remains a challenging problem, and ongoing research is necessary to improve model performance and reliability in clinical settings [159,163,164,165].

In the big-data situation, automatic detection of retinal diseases using deep learning methods such as CNNs and ViTs face a different set of challenges. With a large volume of data, the complexity of the models can increase significantly, leading to longer training times, increased computational resources and potential overfitting. However, the availability of large datasets can also provide opportunities for model optimization and generalization, leading to improved accuracy and reliability of disease detection. Recent studies have demonstrated the effectiveness of deep learning methods in detecting retinal diseases under big-data situations, particularly for common diseases such as diabetic retinopathy and glaucoma [136,164]. Overall, deep learning methods like CNNs and ViTs have shown promising results in detecting retinal diseases under big-data situations. However, the complexity and resource requirements of the models can pose challenges, and ongoing research is necessary to optimize these models and ensure their reliability and effectiveness in clinical settings.

In summary, the following areas remain open research items worth prioritizing:

- Generation of synthetic image data to address the challenge of model overfitting.
- Establishing the right mix and sequence of data pre-processing techniques to enhance image quality.
- Potential impact of ensemble learning for improvement of the performance of CNN architectures.

## 7. Conclusions

This work presented a comprehensive review of the application of deep learning (DL) techniques for retinal disease detection. Several diseases emanating from the eyes, the cardiovascular system or the brain manifests itself through the retina [166,167]. The most prevalent of these are diabetic retinopathy (DR), Age-Related Macular Degeneration (AMD), glaucoma and cardiovascular diseases. If not detected early, these diseases could lead to irreversible loss of vision, putting a heavy burden on individuals, families and already overburdened economies, mostly in undeveloped countries. Fundoscopy and OCT imaging have emerged as the most prevalent noninvasive retinal imaging modalities [168,169]. Manual analysis of retinal images is tedious, time-consuming and prone to subjective assessment, and besides, ophthalmologists who should interpret the images are in short supply and more so in underdeveloped countries [170]. The cited challenges with manual retinal abnormality detection have given rise to the advent of automatic disease classification and segmentation.

The reviews on AMD, glaucoma and multiple disease detection demonstrate that both convolutional neural networks (CNNs) and vision transformers (ViTs) are effective deep learning approaches for retinal disease detection using different imaging modalities and databases.

As noted by [171], CNNs have been widely used for retinal disease detection, and their success can be attributed to their ability to automatically extract features from retinal images without requiring manual feature engineering. CNNs have shown high accuracy in detecting different retinal diseases, including AMD and glaucoma, as well as the ability to detect multiple diseases simultaneously.

Although CNNs have been widely used for retinal disease detection, some authors, for example in [150] concur that there is still limited research on the performance of ViTs for this task. Further research is needed to compare the performance of CNNs and ViTs in terms of accuracy, computational complexity and interpretability. Most of the existing studies focus on the detection of individual diseases, such as AMD, DR and glaucoma, with limited research on the detection of multiple diseases in the same image. Further research is needed to develop deep learning models that can accurately detect multiple diseases in retinal images using both CNNs and ViTs.

Therefore, it is premature to conclude that ViTs are more capable than CNNs for retinal disease detection. Both CNNs and ViTs have their strengths and limitations, and their effectiveness depends on the specific application and the dataset used. Further research is needed to compare the performance of these two deep learning approaches in retinal disease detection using different imaging modalities and datasets [150].

The existing studies mostly use public datasets such as the EyePACS and Messidor datasets, which may not be representative of the general population. Further research is needed to evaluate the performance of deep learning models on diverse datasets and populations using both CNNs and ViTs. Although deep learning models have shown high accuracy in retinal disease detection, there is limited research on their clinical utility and feasibility using both CNNs and ViTs. Further research is needed to investigate the practical application of deep learning models in clinical settings [172].

## Figures and Tables

**Table 1:** Comparison of AO-SLO with FAF, FFA, ICGA and OCT.

Method	Invasive (Y/N)	Transverse Resolution	Field of View	Where Method Is Applied
AO-SLO [39]	N	2.5 μm	1.5°	Observing cones, rods, capillary, vessel and nerve fiber layer
FAF [44]	N	20 μm	50°	CNV, macular edema, retinal pseudodrusen
FFA [45]	Y	20 μm	50°	Aneurysms, tumor, edema, vitreous inflammation
ICGA [46]	Y	20 μm	50°	Exudative AMD, inflammation, edema, tumor, coroidal vasculopathy
OCT [47]	N	20 μm	45°	Vitreoretinal interface disorders, AMD, DR

**Abbreviations:** OCT—Optical Coherence Tomography; ICGA—Indocyanine Green Angiography; FFA—Fluorescein Angiography; FAF—Fundus Autofluorescence; AO-SLO—Adaptive Optics Scanning Laser Ophthalmoscopy.

**Table 2:** Summary of Retinal Image Databases.

Database	Image Count	Resolution	Camera	Field of View	Purpose
DRIVE [50]	40	768 × 564	Canon CR5	45°	Vessel Seg.
STARE [51]	81	700 × 605	TopCon TRV-50	35°	Vessel Seg
ARIA [50]	450	768 × 576	Zeiss FF450+	50°	ONH boundary Seg.
CHASEDB [52]	28	1280 × 960	Nidek NM-200-D	30°	Vessel Seg.
IMAGERET [53]	219	1500 × 1152		50°	DR grading
MESSIDOR [54]	1200	1440 × 960	TopCon TRC	45°	DR Grading
MESSIDOR-2 [55]	1200	Various res	Topcon TRC NWC	45°	DR Grading
e-Ophtha [56]	434	Various res	Various	45°	DR Screening
DIARET DB1 [57]	89	Various res	Various	50°	DR Grading
APTOS [58]	3662	Various res	Various		DR Grading
AREDS [59]	120,656	Various res	Carl Zeiss AG	30°	AMD
ORIGA [60]	650	3072 × 2048			Disc & Cup Seg.
SCES [76]	1676	3888 × 2592			Glaucoma detection
ACRIMA [62]	705	2048 × 1536	Topcon TRC	35°	Glaucoma detection
RIM-ONE [63]	159	2144 × 1424	Kowa WX 3D	20° (hor), 27° (vert)	Glaucoma detection
LAG [64]	11,760	1977 × 2594	Various		Glaucoma detection
OCT2017 [65]	84,484				Multidisease detection
SERI DB [66]	4096	1024 × 512	Carl Zeiss Meditec Inc.		DME classification
ODIR [78]	6426	Various res	Various		Multidisease detection
OIA-ODIR [68]	10,000	Various res	Various		Multidisease detection
ROC [69]	100	768 × 576	TopCon NW100	45°	MA detection
IOSTAR [70]	30	1024 × 1024	SLO	45°	Vessel Seg.
Kaggle [73]	88,702	Various res	Various	Various	DR Grading
REVIEW [71]	16	3584 × 2438 (HRIS)	Canon 60 UV	60° (HRIS)	Vessel Seg.
DR HAGIS [72]	39	Various res	Various	Various	Multidisease detection
TROPIC [77]	130	640 × 480	RetCam130	130°	ROP
RetCam3 [75]	80	640 × 480	RetCam3	120°	ROP

**Table 3:** Summary of Deep Learning Methods for DR Classification.

Reference	Network	Dataset	Accuracy	Specificity	Sensitivity	AUC
[55]	AlexNet	Messidor-2		87.0%	96.8%	
[80]	CNN	EyePACS		66.6%	96.2%	0.946
[81]	CNN	EyePACS	86.1%	93.81%	73.24%	0.92
[82]	ConvNet	EyePACS, e-optha, DiaretDB1				0.954, 0.949, 0.955
[83]	CKML, VNXK	Messidor, EyePACS	0.897, 0.893	0.900, 0.892	0.893, 0.900	0.891, 0.887
[94]	DCNN	EyePACS	0.973, 0.959	0.863, 0.898	0.9687, 0.9687	
[95]	AttenNet (DenseNet 169)	Z109 (public), B28K (private)		1.000, 0.915	1.000, 0.924	
[6]	Ensemble CNN	EyePACS	0.808	0.867	0.515	
[96]	Inception-V3	Private	0.8791	0.9150	0.844	0.935
[87]	VGG-16, VGG-19	EyePACS	0.820	0.82	0.800	
[88]	EfficientNet	APTOS				0.935 (Kappa)
[89]	DenseNet-121	APTOS	0.949	0.971	0.926	0.88 (Kappa)
[97]	Ensemble DCNN					
[98]	DCNN	MESSIDOR-2	0.941	0.957	0.927	0.969
[99]	ViT-DR	MESSIODOR-2, e-ophtha, APTOS, IDRiD				0.956, 0.975, 0.946, 0.924
[100]	ViT	MESSIDOR-2, e-ophtha, APTOS				0.956, 0.977, 0.947
[101]	Res-ViT	MESSIDOR-2, APTOS	0.893, −			−, 0.981
[102]	ensemble-ViT	MESSIDOR-2, APTOS	−, 0.912			0.977, −
[90]	DCNN	EyePACS	0.757			

**Table 4:** Summary of AMD Detection methods.

Reference	Network	Dataset	Acc	Sp	Sn	AUC
[104]	DCNN + SVM	AREDS	0.950	0.956	0.964	
[79]	VGG16	AREDS	0.925			
[106]	CNN	Private	0.955	0.935	0.9643	
[107]	DCNN	Private				0.998
[108]	Ensemble	AREDS		0.842	0.943	
[103]	DCNN	AREDS	0.950	0.956	0.964	
[18]	DCNN	Private	0.966	0.974	0.978	0.999
[105]	DCNN	Private	1.000, 0.996, 0.998	1.000, 0.992, 0.996	1.000 *, 1.000 **, 1.000 ***	
[110]	ViT	MESSIDOR, APTOS	−, 0.913			0.963, −
[111]	ViT	AREDS	0.994			0.993
[109]	AMDOCT-Net	Private	0.991, 0.957	1.000, 0.920	0.982 +, 0.993 ++	

* Classifying healthy and wet AMD. ** Classifying healthy and dry AMD. *** Classifying healthy and DME. + AMDOCT without cropping. ++ AMDOCT with cropping. Acc: Accuracy. Sp: Specificity. Sn: Sensitivity.

**Table 5:** Summary of DL Methods for Glaucoma Detection.

Reference	Network	Dataset	Acc	Sp	Sn	AUC
[112]	6L CNN	ORIGA SCES				0.831, 0.887
[23]	22L DCNN	LabelMe		0.920	0.956	0.986
[113]	MB-NN	Private	0.915	0.909	0.923	
[122]	DCNN	RIM-ONE	0.894			
[114]	DNet-201	ACRIMA	0.970	1.000	0.941	0.971
[121]	GoogleNet ResNet-50	RIM-ONE	0.910, 0.900	0.990, 0.940	0.170, 0.420	0.910 *, 0.840 *
	GoogleNet ResNet	RIM-ONE	0.850, 0.860	0.910, 0.930	0.290, 0.210	0.750 **, 0.740 **
[64]	AG-CNN	LAG	0.962	0.967	0.954	0.983
[116]	ResNet-50	DRISHTI-GS1, RIM-ONE V3	0.987, 0.961			
[117]	ViT	ORIGA, RIM-ONE v3		0.912, 0.957	0.923, 0.941	
[118]	ViT	ORIGA				0.960
[120]	ViT	ORIGA	0.737			0.964
[119]	ViT	RIGA	0.902			0.975
[115]	ResNet-50	Private	0.970			

* Performance on early glaucoma detection. ** Performance on advanced glaucoma detection. Acc: Accuracy. Sp: Specificity. Sn: Sensitivity.

**Table 6:** Summary of Deep Learning Methods for the Detection of Multiple Retinal Diseases.

Reference	Network	Dataset	Acc	Sp	Sn	AUC
[127]	Ensemble ELPPJD	Private	0.886	0.8859	0.886	
[124]	OpticNet-71	OCT2017	0.998	0.999	0.998	
[125]	ResNet50	Private	0.973	0.985	0.963	
[126]	ResNet101	FAF images	0.950	0.983	0.935	0.999
[3]	AOCT-NET	SERI DB	0.971	0.993	0.971	0.995
[14]	VGG16	ODIR	0.891			0.689
[123]	Ensemble (ResNet-152)	Private	0.989	0.996	0.989	
[67]	FCL-EfficientNet-B3	OIA-ODIR	* 0.994	** 0.991	*** 0.995	
[128]	Inception-v3	IDRiD, MESSIDOR	0.954, 0.965			
[129]	ViT	IDRiD, Messidor-2, APTOS	0.9847, 0.9667, 0.9576			
[130]	Att-ViT	MESSIDOR-2, EYEPACS				
[131]	RetinaNet-ViT	IDRiD, MESSIDOR-2	−, 0.971	0.966, −	0.944, −	
[132]	Att-ViT	KDD	0.897			

* Performance for AMD class. ** Performance for cataract class. *** Performance for glaucoma class. Acc: Accuracy. Sp: Specificity. Sn: Sensitivity.

## Data Availability

The data used in the paper are publicly available.

## Abbreviations

The following abbreviations are used in this manuscript:

DCNNs	Deep Convolutional Neural Networks
CAD	Computer-Aided Diagnosis
CNV	Choroidal Neovascularization
AMD	Age-related Macular Degeneration
DME	Diabetic Macula Edema
DR	Diabetic Retinopathy
OCT	Optical Coherence Tomography
ML	Machine Learning
DL	Deep Learning
AI	Artificial Intelligence
ViT	Vision Transformer
